# Supplementary material for: A Critical View of the Application of the APEX Software (Aqueous Photochemistry of Environmentally-Occurring Xenobiotics) to Predict Photoreaction Kinetics in Surface Freshwaters
Source: Molecules. 2019 Dec 18;25(1):9. doi: 10.3390/molecules25010009 (PMC7017383; doi:10.3390/molecules25010009)
Supplement: Supplementary file 1 [file molecules-25-00009-s001.zip › 3.2.4_gcc-4.4.0.zip!/3.2.4_gcc-4.4.0/3.2.4_gcc-4.4.0/doc/octave/html/interpreter/Tips-and-Standards.html]

Tips and Standards - Untitled


Next: Contributing Guidelines,
Previous: Test and Demo Functions,
Up: Top


---

## Appendix C Tips and Standards

This chapter describes no additional features of Octave. Instead it
gives advice on making effective use of the features described in the
previous chapters.

- Style Tips: Writing clean and robust programs.- Coding Tips: Making code run faster.- Comment Tips: Conventions for writing comments.- Function Headers: Standard headers for functions.- Documentation Tips: Writing readable documentation strings.
